# Association between thyroid function and nonalcoholic fatty liver disease: a dose-response meta-analysis

**DOI:** 10.3389/fendo.2024.1399517

**Published:** 2024-06-25

**Authors:** Liu-lan Xiang, Yu-tian Cao, Jing Sun, Rui-han Li, Fang Qi, Yu-juan Zhang, Wen-hui Zhang, Lou Yan, Xi-qiao Zhou

**Affiliations:** ^1^ Department of Endocrinology, Affiliated Hospital of Nanjing University of Chinese Medicine, Jiangsu Province Hospital of Chinese Medicine, Nanjing, China; ^2^ The First Clinical Medical College of Nanjing University of Chinese Medicine, Nanjing, China

**Keywords:** thyroid function, nonalcoholic fatty liver disease, dose-response, meta - analysis, systematic evaluation

## Abstract

**Background:**

Thyroid hormones (THs) have been found that it is closely associated with the onset and progression of non-alcoholic fatty liver disease (NAFLD). However, the current study could not verify the intrinsic relationship between thyroid hormones and NAFLD, which requires further research.

**Methods:**

The searches of studies reported both TH level in serum and NAFLD were performed in PubMed, Web of Science, Cochrane Library, and Embase databases. We combined an overall meta-analysis with a dose-response meta-analysis to assess the correlation and dose-response relationship between thyroid function levels and the risk of NAFLD.

**Results:**

Overall, 10 studies were included with a total of 38,425 individuals. We found that the non-linear dose-response model showed that for every 1 ng/dL increase in FT4, the risk of NAFLD was reduced by 10.56% (p=0.003). The odds ratios (ORs) for NAFLD with high free triiodothyronine (FT3) exposure compared to those with low FT3 were 1.580 (95% CI 1.370 to 1.830, I2 = 0.0%, p<0.001) in the overall meta-analysis. The continuous variable meta-analysis indicated that individuals with high levels of TSH (SMD=1.32, 95% CI 0.660 to 1.970, p<0.001) had significantly higher levels of liver fibrosis than those with low levels.

**Conclusions:**

Our findings only validate that there is a correlation between the occurrence of NAFLD and abnormal levels of THs, and it is expected that more observational studies will still be conducted in the future to further demonstrate the relationship between thyroid hormones and NAFLD.

**Trial registration:**

Registered number in PROSPERO: CRD42023405052.

## Introduction

Nonalcoholic fatty liver disease (NAFLD) is the most prevalent liver disease, affecting about 25% population worldwide (1). From a pathological perspective, NAFLD includes liver injuries of varying severity degrees and the resulting liver fibrosis (2). Of these, hepatic steatosis (fatty liver) alone is defined as Nonalcoholic fatty liver (NAFL), while a more severe liver damage with apparent inflammation in hepatocytes is known as Nonalcoholic steatohepatitis (NASH) (2). NASH is not only known to be the early stage of multiple irreverseble liver diseases, such as cirrhosis, liver failure and hepatocellular carcinoma, but also strongly associated with adverse outcomes in non-liver diseases like cardiovascular disease and malignant disease (3). Therefore, before developing to inrreverseble status, it still warrants the attention of assessing other risk factors relating to NAFLD progression while still awaiting the effective therapies of NAFLD.

In the human body, thyroid hormones (THs), including thyroxine (T4), triiodothyronine (T3) and thyroid-stimulating hormone (TSH), are all essential hormones in mediating the synthesis and metabolism of fatty acids and cholesterol in the liver (4). Their participations in regulating the whole body’s metabolism are critical to maintain specific functions of several tissues and cell types (5). Imbalances of these hormones may result in overactive metabolism due to excess thyroid hormones (hyperthyroidism), or underactive activity due to deficient thyroid hormones (hypothyroidism). Depending on the severity of this imbalance, both types of THs disorder can be divided into clinical or subclinical status, with the latter being more common (6). Subclinical hypothyroidism (SH) has been considered as a risk factor for NAFLD. Furthermore, the possibility of liver steatosis improvement through SH treatment is also raised (7). As the liver and thyroid are closely associated with thyroid hormone, its important role in *de novo* lipogenesis (DNL), beta-oxidation (FAO), cholesterol metabolism and carbohydrate metabolism has led to a number of animal studies and clinical trials investigating TH analogues and TR agonists as potential therapies for NAFLD and hyperlipidemia (8). However, the relevant evidence above the clinical level is incomplete and still needs to be further supplemented.

So far, four published meta-analyses (9–12) have explored the relationship between thyroid and NAFLD. Interestingly, their conclusions are mutually controversial. Gu (9) suggested that the level of TSH might be a risk factor for the development and progression of NAFLD, which was independent of other THs levels; He et al. (10) found that both subclinical and overt hypothyroid patients were at higher risks of NAFLD than those with normal thyroid function. On the other hand, Mantovani et al (11) suggested there was a significant and independent association between primary hypothyroidism and the incidence and severity of NAFLD, but whether subclinical hypothyroidism independently predicts the progression of NAFLD remain uncertain. However, analysis conducted by Jaruvongvanich et al (12). showed there were no significant associations between NAFLD and subclinical, overt or overall hypothyroidism. They also found that the THs level between participants with and without NAFLD had no significant differences.

Although the relationship between the thyroid and NAFLD is widely discussed, it is still not clear if thyroid hormone levels associate with the development of NAFLD. Therefore, we conducted this dose-response meta-analysis of observational studies (incuding cohort and case-control studies) to further investigate on the dosage-dependent correlationship between THs levels, thyroid diseases and NAFLD.

## Methods

### Search strategy and selection criteria

This meta-analysis was conducted following the Meta-analysis of Observational Studies in Epidemiology (MOOSE) reporting guideline (13) and the Preferred Reporting Items for a Systematic Review and Meta-analysis (PRISMA) guideline (Additional file 1) (14). The protocol for this meta-analysis was registered with PROSPERO (CRD42023405052).

We have searched the PubMed, Web of Science, Cochrane Library, and Embase databases for relevant articles published up to February 16, 2023. Our search strategy (Hyperthyroidism OR Hypothyroidism OR Thyroid Hormones OR Thyrotropin OR Thyroid Function Tests OR subclinical hypothyroidism OR subclinical hyperthyroidism OR subclinical thyroid dysfunction) consisted of MeSH terms and entry terms with no restrictions on the language of the articles. Additionally, bibliographies of related articles and current review articles were manually screened for potentially relevant articles.

The inclusion criteria were as follows: (1) cohort or case-control study;(2) the thyroid function level and the risk of NAFLD or NAFLD liver fibrosis index were displayed;(3) providing sufficient data to report odds ratios (ORs), relative risks (RRs), or hazard ratios (HRs) with 95% confidence intervals (95% CIs) to assess the association between thyroid function and NAFLD.

The exclusion criteria were: (1) not conducted with humans; (2) narrative and/or systematic reviews, commentaries, case reports, cross-sectional studies; (3) no relevant data were reported or could not be extracted.

### Data collection and extraction

Studies in the database are administered and duplicate articles were removed using EndNote X9 software. The articles were screened by our two co-workers (Fang Qi & Wen-hui Zhang) independently, following the inclusion and exclusion criteria above. Disagreements were discussed with another co-author (Yu-tian Cao) and subsequently solved via consensus. Liu-lan Xiang and Yu-juan Zhang extracted data along with relevant information from the selected articles and entered it into a standard form. The contents include: general information of the articles (first author’s name, the publication year and region), participants information (age, gender, number of subjects, number of NAFLD cases and mean/median/range of thyroid function levels, BMI, or liver fibrosis index), study design (type of study, year of follow-up, source of cases).

### Quality assessment

According to the quality evaluation criteria of observational studies, the Newcastle-Ottawa Scale (NOS) was used to evaluate cohort studies and case control studies (15–24). The NOS evaluates the quality of research from three aspects: the selection of the study groups; the comparability of the groups; and the ascertainment of either the exposure or outcome of interest for case control or cohort studies, respectively (Additional file 2). The quality of the study was assessed as follows: low quality = 0–3 stars; moderate quality = 4–6 stars; and high quality = 7–9 stars. If the answer is “unclear” or “no”, the item score is “0”. If the answer is “yes”, the score of the item is “1” (25). The quality of eligible articles were independently assessed by two investigators (Liu-lan Xiang and Yu-juan Zhang). Disagreements were discussed and solved by the third investigator (Yu-tian Cao).

### Data synthesis and data analysis

To exclude potential heterogeneity, we estimated combined risk ratios (ORs) and corresponding 95% confidence intervals (CIs) by using a random effects model (26). The standardized mean difference (SMD) and 95% confidence interval (95% CI) of liver Cirrhosis index and controls were calculated and evaluated. Where articles provided studies with only median, range and/or interquartile spacing, we used the method proposed by Cai et al. (27) and McGrath et al. (28) to estimate the sample mean and SD.

We also used prespecified subgroup analysis to identify the main sources of heterogeneity and to assess the robustness of the pooled results. The subgroup were defined as follows: the age of participants (≥ 60 years, < 60 years, not reported; ≥ 18 years, < 18 years in NAFLD liver fibrosis analysis), sex (women ≥ 50%, women < 50%, not reported), BMI (normal weight, overweight or obese, not reported), follow-up years (≥ 5 years, <5 years), study location (Asia, others), sample size (>median, ≤ median), and study type (case control study, cohort study) and the quality of study according to the NOS (7, 8, and 9 stars).

Futhermore, in order to establish an “average” dose-response relationship between thyroid function/hormone levels and risk of NAFLD based on the data of all available studies, the robust-error meta-regression (REMR) model was employed in this study (29). In regression model (30), non-linear curves were fitted by restricted cubic spline. The Wald test was used to test for non-linearity by assuming that the coefficient on the non-linear term was zero.

Statistical heterogeneity among the studies was quantified using the Cochrane Q statistic (p<0.100 was considered indicative of statistically significant heterogeneity) and the I^2^ statistic (ranges from 0% to 100% with lower values representing less heterogeneity) (31).

Besides, sensitivity analysis was performed to evaluate whether results from the individual study could substantially influence the pooled results of thyroid function levels and NAFLD. The funnel plot asymmetry test for measuring publication bias was not used because less than 10 studies were included in each analysis (32).

All analyses were conducted using Stata MP 16 (StataCorp, College Station, TX). A two-tailed P<0.050 was considered statistically significant.

## Results

### Search and selection result

**Figure 1:**
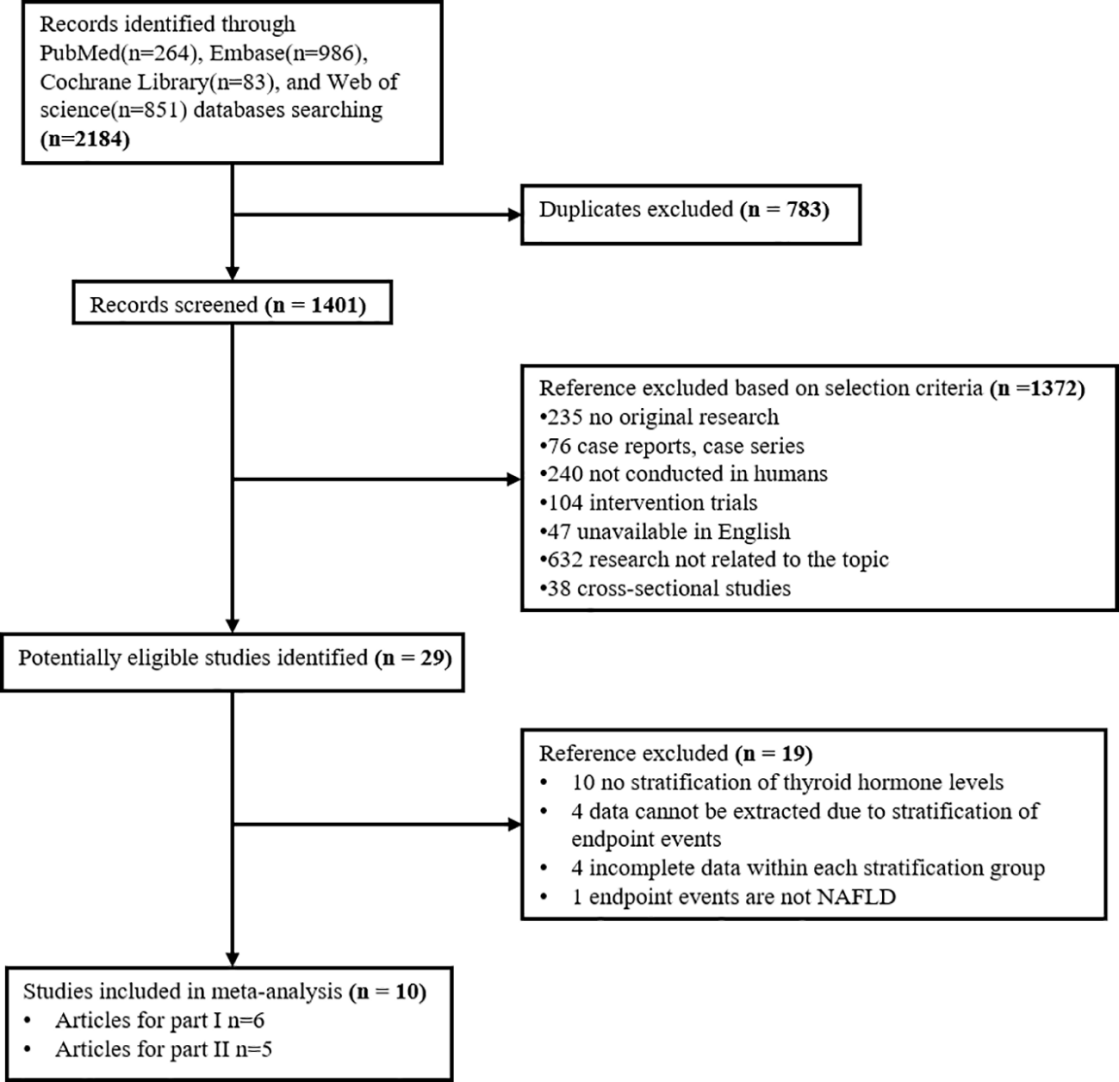
Flow diagram of study selection process.

Our search in four databases: PubMed, Embase, Cochrane Library, and Web of science, obtained 2184 records. At last, 10 studies for dose-response meta-analysis were identified. Of these, six articles were included to explore the relationship between thyroid hormone levels and the risk of NAFLD; five (with 1 duplicate) were selected to investigate the relationship between thyroid hormone levels and the fibrosis level in NAFLD (**Figure 1**).

### Characteristics of included studies

6 publications involving 2 prospective cohort studies, 3 retrospective cohort studies and 1 case-control studies were included in our meta-analysis of thyroid hormone levels and the risk of NAFLD. Among these, 1 study was from European population and others were from Asia. The total sample population was 35,917 individuals and the average follow-up time was 6 years (**Table 1-A**). The Newcastle-Ottawa scale result showed that all 6 studies were of high quality with NOS scores of 7 stars or more.

**Table 1-A:** Study characteristics of 6 Observational study (Thyroid hormones and NAFLD risk) included in the meta-analysis.

Author, Study	Study type	Region;years	Source of cases	Outcomes; diagnostic method	cases/total no. of subjects	Age, y	Female, %	BMI,kg/m2	Thyroid function group	NOS
**Bano et al.** (15)	prospective cohort study	Netherlands;1990-2006	The Rotterdam Study (RS)	NAFLD;abdominal ultrasonographies;FIL	1217/9419	64.7±9.7	5321(56.5)	27.2±4.2	Hypothyroidism	9
									Euthyroidism	
									Hyperthyroidism	
**Lee, Cho et al.** (16)	retrospective cohort study	Korea;2012-2018	participants in a medical health check-up program at the Health Promotion Center of Kangbuk Samsung Hospital	NAFLD;abdominal ultrasonography	2348/18,544	39.2±5.9	647(28)	24.2±2.4	Euthyroidism	9
									Subclinical Hypothyroidism	
									Overt Hypothyroidism	
**Wang, Niu et al.** (17)	Case–Control Study	China;2014.9-2019.9	who were hospitalized in the Department of Endocrinology of the First Hospital of Lanzhou University	NAFLD;bdominal ultrasound	212/400	63 (53~73)	107(50.5)	22.83±3.48	Thyroid Autoimmunity Positive	7
									Thyroid Autoimmune Negative	
**Gu, Wu et al.** (18)	prospective dynamic cohort study	China;2013-2019	The Tianjin Chronic Low-grade Systemic Inflammation and Health (TCLSIH) Cohort Study	NAFLD;TOSHIBA SSA-660A ultrasound machine ultrasonography	1675/6462	51.5±7.90	621(37.1)	25.3±2.65	TSH:0.55–1.35(mIU/L); FT3:227–293(pg/dL); FT4 :0.89–1.07(ng/dL)	8
									TSH:1.36–1.84(mIU/L); FT3:294–322(pg/dL); FT4:1.08–1.18(ng/dL)	
									TSH:1.85–2.52(mIU/L); FT3:323–349(pg/dL); FT4:1.19–1.31(ng/dL)	
									TSH:2.53–4.78(mIU/L); FT3:350–422(pg/dL); FT4:1.32–1.76(ng/dL)	
**Zhang, Li et al.** (19)	retrospective cohort study	China;2016-2018	clinical analysis of 980 T2DM patients recruited and admitted, from March 2016 to December 2018, to the department of endocrinology of the First Affiliated Hospital of Anhui University of Traditional Chinese Medicine	NAFLD;abdominal color ultrasound	346/980	58.5(52–66)	184(53)	26.25±3.37	TSH:2.5104±1.1098(uIU/L); FT3:3.95±0.50(pmol/L); FT4:12.55±1.40(pmol/L)	7
									TSH:2.0308(1.3439–2.7457)(uIU/L); FT3:4.06±0.47(pmol/L); FT4:13.12±2.49(pmol/L)	
**Nien, Sheu et al.** (20)	cohort study	Taiwan,China;one-year	These 122 subjects visited the clinics	fatty liver;abdominal ultrasonography(Toshiba SSA-320A, SSA-660A, or Aplio 300,)	95/112	NA	NA	NA	TSH:1.36±0.78(μU/mL); FT4:1.05±0.13(ng/dL)	6
									TSH:2.01±0.91(μU/mL); FT4:1.04±0.09(ng/dL)	
									TSH:1.89±1.31(μU/mL); FT4:1.02±0.12(ng/dL)	

NA means not applicable.

In addition, we selected one study (20) from the 6 studies above and identified other 4 studies (2 studies in Asia, one in Africa and one from the Turkish population) to conduct the meta-analysis of thyroid hormones and liver fibrosis in NAFLD. These 4 studies also scored more than 6 stars in NOS. The sample population of was 2,508 participants in all with a follow-up period of 1–10 years (**Table 1-B**).

**Table 1-B:** Study characteristics of 5 Observational study (Thyroid hormones and NAFLD liver cirrhosis index) included in the meta-analysis(One article was used in two studies).

Author, Study	Study type	Region;years	Source of cases	Thyroid function group	Outcomes;diagnostic method	total no. of subjects	Mean±SD	cases no. of subjects	Age, y	Female, %	BMI,kg/m2	NOS
**Türker, Oral et al.** (21)	retrospective cohort study	Turkey;2010.1-2020.1	data from living donor candidates who underwent liver biopsies and patients with NASH-associated cirrhosis at the Gastroenterology and Internal Medicine Department, Florence Nightingale Hospital, Demiroglu Bilim University	TSH:2.72±8.13(mIU/L); FT3:4.11±22(mIU/L); FT4:8.14±7.10(mIU/L)	liver Cirrhosis index;FIB-4	436	0.6±0.2	142	34.08±9.00	NA	24.70±3.30	8
				TSH:2.00±1.20(mIU/L); FT3:4.82±1.56(mIU/L); FT4:3.34±0.83(mIU/L)			0.6±0.2	226	34.25±8.7	NA	27.20±4.00	
				TSH:2.58±2.73(mIU/L); FT3:13.74±10.28(mIU/L); FT4:13.41±6.32(mIU/L)			6.4±4.0	68	59.93±6.7	NA	30.30±5.11	
**Nien, Sheu et al.** (20)	cohort study	Taiwan,China;one-year	These 122 subjects visited the clinics	TSH:1.36±0.78(μU/mL); FT4:1.05±0.13(ng/dL)	liver Cirrhosis index;FIB-4	122	0.927±0.397	21	49.0±10.0	3(12.5)	21.6±1.9	6
				TSH:2.01±0.91(μU/mL); FT4:1.04±0.09(ng/dL)			0.956±0.389	39	50.6±10.0	25(64.1)	24.9±1.6	
				TSH:1.89±1.31(μU/mL); FT4:1.02±0.12(ng/dL)			0.850±0.410	62	48.9±10.8	35(56.45)	29.4±4.1	
**Du, Chai et al.** (22)	case-control study	China;2014.12-2019.10	Cases with T2DM who were admitted to the Endocrinology Department of Peking University International Hospital	TSH:2.01(1.34, 2.97)(mU/L); FT3:4.50±1.01(pmol/L); FT4:16.69±10.95(pmol/L)	liver Cirrhosis index;FIB-4	1422	1.068(0.774, 1.465)	586	57.28±14.13	288(38.91)	25.38±4.04	8
				TSH:2.05(1.29, 2.87)(mU/L); FT3:4.21±1.50(pmol/L); FT4:16.42±7.46(pmol/L)			1.162(0.857, 1.590)	836	53.07±13.32	308(36.84)	26.32±3.68	
**Naguib, Fayed et al.** (23)	case-control study	Egypt;NA	who had NAFLD and were evaluated in the Hepatology Outpatient Clinics in Alexandria University Hospitals.	TSH:3.3±1.2(μIU/ml); FT4:1 ±0.3(ng/dl)	liver Cirrhosis index;FIB-4	100	2.4 ±1.9	50	47.9±3	28 (56)	31.6 ±4.7	6
				TSH:2.2±0.8(μIU/ml); FT4:1.2 ±0.3(ng/dl)			0.7 ±0.3	50	45.7±4	27 (54)	25.4 ±2.3	
**Choi, Yi et al.** (24)	retrospective cohort study	Korea;2015.1-2019.12	10 centers in Korea: Chungnam National University Hospital, Inje University Haeundae Paik Hospital, Chung-Ang University Hospital, Jeonbuk National University Hospital, Kyungpook National University Children's Hospital, Korea University Anam Hospital, Soonchunhyang University Bucheon Hospital, Nowon Eulji Medical Center, Keimyung University Dongsan Medical Center, and Inje University Ilsan Paik Hospital.	Euthyroidism	liver Cirrhosis index;APRI score	428	0.67 ± 0.50	370	12.16±2.97	110(29.7)	28.04±5.00	6
				SH			1.06 ± 0.89	58	12.19±3.31	16(27.6)	28.40±4.84	

NA means not applicable.

### Thyroid hormone levels and risk of NAFLD

#### High versus Low

**Figure 2:**
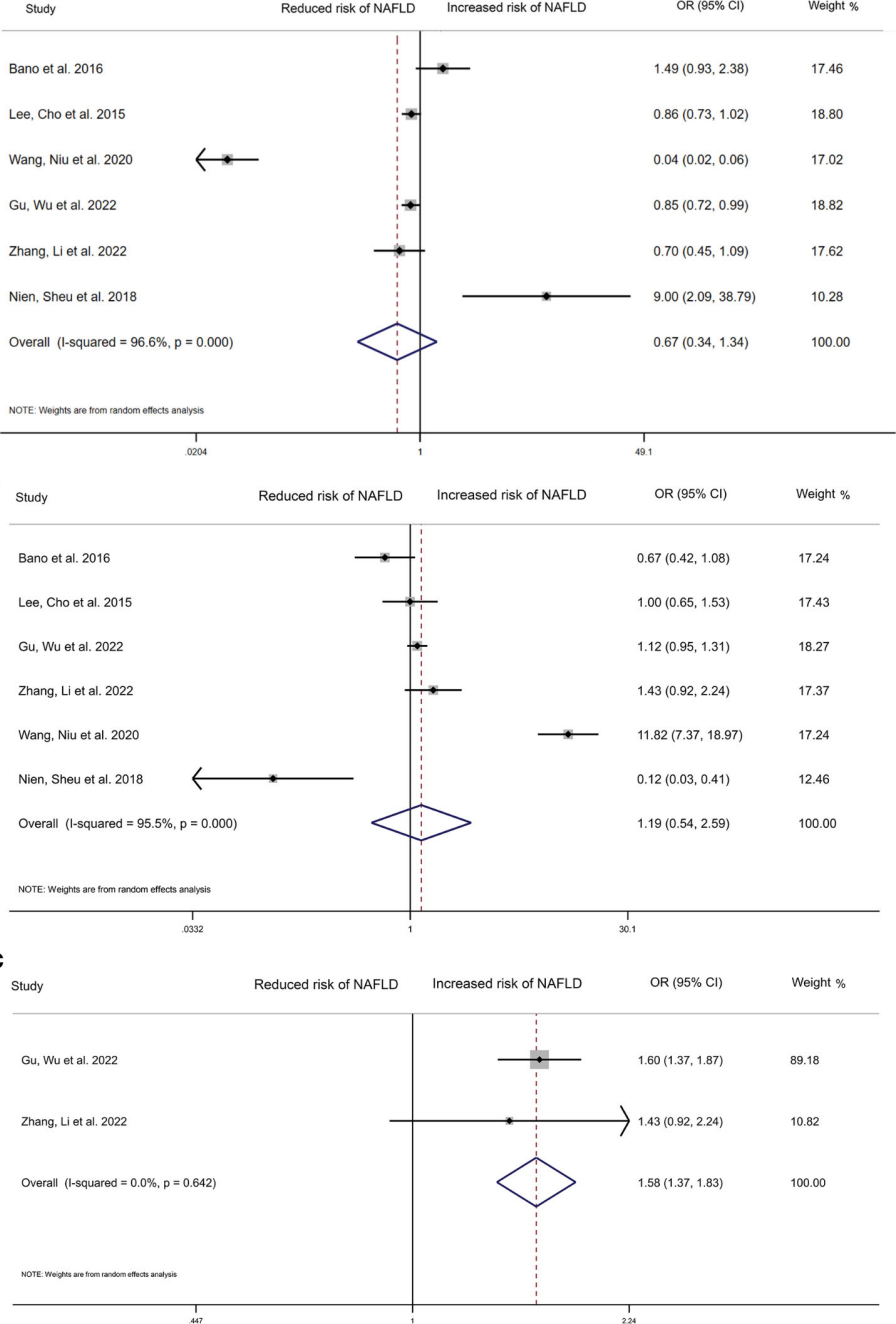
Forest plot of the risk of NAFLD associated with Thyroid hormones. **(A)** Association between TSH and risk of NAFLD. **(B)** Association between FT4 and risk of NAFLD. **(C)** Association between FT3 and risk of NAFLD. The hollow diamond represents the merged OR. The error bar represents a 95% confidence interval.

We compared the highest and lowest level of thyroid hormones to analyse the relationship between TSH, FT4 and the risk of NAFLD in six studies (35,917 participants). High serum TSH level (OR=0.670, 95%CI 0.336–1.341, I^2^ = 96.6%, P=0.259), and high FT4 (OR=1.190, 95%CI 0.540 to 2.590, I^2^ = 95.5%, p=0.666) were found to not be significantly related with NAFLD risk (**Figures 2A, B**). For FT3, we have pooled the effects of 2 articles (7,442 participants) and found a significant relationship between high serum FT3 concentrations (OR=1.580, 95%CI 1.370 to 1.830, I^2^ = 0.0%, p<0.001, **Figure 2C**) and incidence of NAFLD.

#### Dose-response analysis

**Figure 3:**
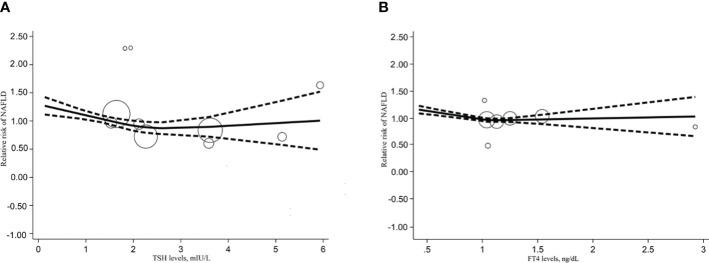
Dose response relationship between TSH, FT4 level and NAFLD risk. **(A)** Dose-response relationship between TSH levels and relative risk of NAFLD. **(B)** Dose-response relationship between FT4 levels and relative risk of NAFLD.

We also performed a dose-response meta-analysis of the included 6 studies with the REMR model to further illustrate the possible link between different level of THs and the risk of NAFLD. There seemed to exist a non-linear dose-response association between the TSH level and the relative risk of NAFLD, which presented as a “U” shape (**Figure 3A**). What’s more, FT4 level showed a significant negative non-linear correlation with the risk of NAFLD (p=0.003) (**Figure 3B**). After fitting the data with the three-section RCS, we found that when FT4 levels were above 1.019 ng/dL, every 1 ng/dL increase in FT4 would lead to a 10.56% reduction in the relative risk of NAFLD (Additional file 4). Regretfully, this result only disaplayed a trend since it was not statistically significant (p=0.652). As for the association between the FT3 level and the risk of NAFLD, the limited sample size was not enough to perform the dose-response meta-analysis.

### Thyroid hormone levels and extent of liver fibrosis

#### High versus Low

**Figure 4:**
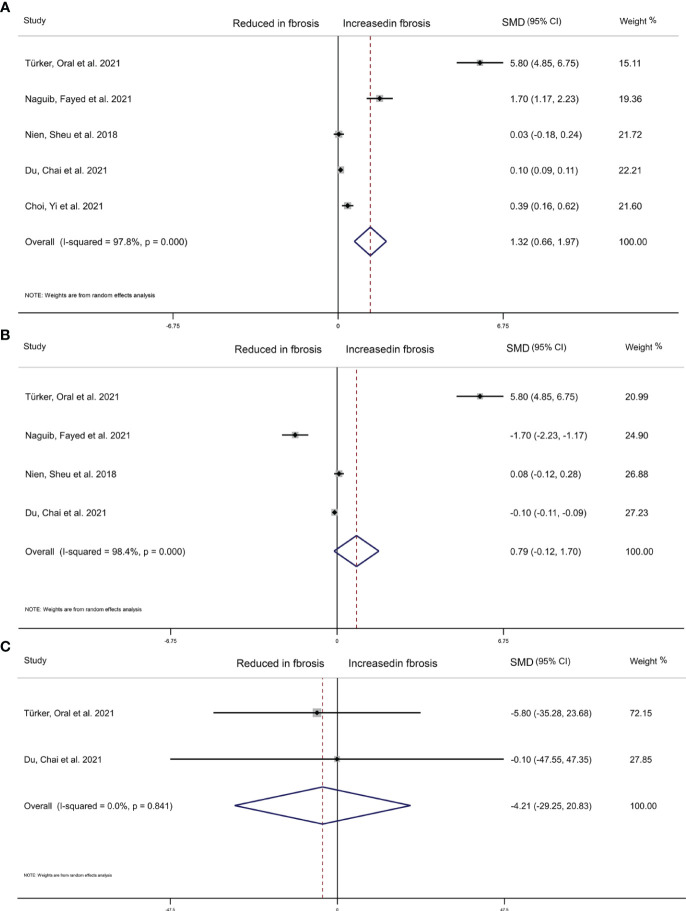
Forest plot of NAFLD liver cirrhosis index associated with Thyroid hormones. **(A)** Association between TSH and NAFLD liver cirrhosis index. **(B)** Association between FT4 and NAFLD liver cirrhosis index. **(C)** Association between FT3 and NAFLD liver cirrhosis index. The hollow diamond represents the merged SMD. The error bar represents a 95% confidence interval.

We collected 5 articles with 2,508 participants to explore the relationship between THs level and the extent of liver fibrosis. The result of random-effects model indicated that the estimated value of the combined effect size of the SMD point was 1.320 (95%CI 0.660 to 1.970, p<0.001) related to liver fibrosis and TSH level, suggesting that the incidence of liver fibrosis in high TSH level group was significantly higher than in the low TSH group (**Figure 4A**). Additionally, as continuous variables, FT4 (SMD=0.790, 95%CI -0.120 to 1.70, p=0.090), or FT3 (SMD=-4.210, 95%CI -29.250 to 20.830, p=0.742) did not significantly correlate with the extent of liver fibrosis (**Figures 4B, C**).

#### Dose-response analysis

**Figure 5:**
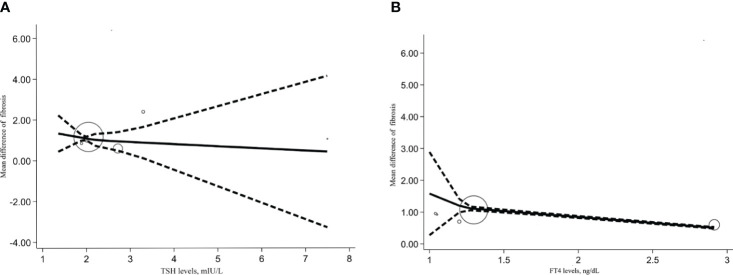
Dose response relationship between TSH, FT4 level and NAFLD liver cirrhosis index. **(A)** Dose-response relationship between TSH levels and NAFLD liver cirrhosis index. **(B)** Dose-response relationship between FT4 levels and NAFLD liver cirrhosis index.

We also conducted a dose-response meta-analysis of five studies using the REMR model. There was no dose-response correlation between TSH levels (p=0.273), serum FT4 levels (p=0.391) and the degree of hepatic fibrosis (**Figures 5A, B**).

### Subgroup and sensitivity analyses

**Figure 6:**
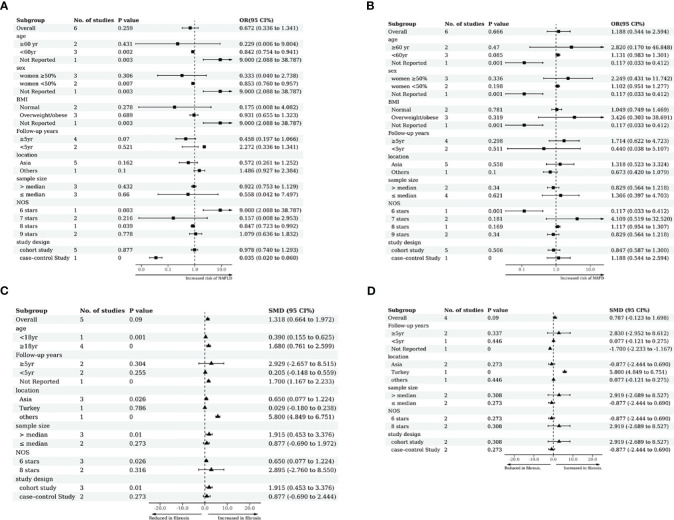
Subgroup analyses. **(A)** Subgroup analyses of TSH levels and relative risk of NAFLD. **(B)** Subgroup analyses of FT4 levels and relative risk of NAFLD. **(C)** Subgroup analysis of TSH levels versus NAFLD liver cirrhosis index. **(D)** Subgroup analysis of FT4 levels versus NAFLD liver cirrhosis index.

We conducted subgroup analyses based on age, gender, study site, BMI, years of follow-up, sample size, quality scores and study design. As shown in **Figure 6A**, subgroup analysis of the TSH-NAFLD correlation in studies of individuals under 60 years old showed a significant negative correlation between high TSH levels and NAFLD (OR=0.842, 95%CI 0.754 to 0.941, p=0.002). High TSH concentrations in subgroups with less than 50% female representation (OR=0.853, 95%CI 0.760 to 0.957, p=0.007) were significantly associated with low risk of NAFLD (**Figure 6A**). There was a significant correlation between high TSH level and liver fibrosis in the cohorts aged >18 (SMD=1.680, 95%CI 0.761 to 2.599, P<0.001) (**Figure 6C**). High TSH level was also remarkably associated with liver fibrosis in NAFLD in cohorts from an Asian population (SMD=0.650, 95%CI 0.077 to 1.224, P=0.026) (**Figure 6C**). Furthermore, high TSH level was also significantly associated with liver fibrosis in cohorts with sample sizes above the median (SMD=1.915, 95%CI 0.453 to 3.376, P=0.010) and in subgroups of cohort studies (SMD=1.915, 95%CI 0.453 to 3.376, P=0.010) (**Figure 6C**). The subgroup analysis of the association between serum FT4, the risk of NAFLD and liver fibrosis extent obtained no significant results (**Figures 6B, D**). The results were stable by omitting one study at once (Additional file 5). According to the guidelines, meta-regression and publication bias were not conducted after considering the limited numbers of included studies (N<10).

## Discussion

The purpose of this study was to evaluate the relationship between the thyroid function and NAFLD risk as well as the NAFLD-related fibrosis markers. After finishing this meta-analysis of 10 published observational studies, we found that high levels of FT3 are significantly associated with a high risk of NAFLD. Also, there was a significant nonlinear inverse association between elevated FT4 and incidence of NAFLD. What’s more, we found that indicators of liver fibrosis were significantly higher in individuals with high TSH hormone levels.

Thyroid hormones are known to play significant role in regulating the whole-body energy expenditure as well as hepatic lipid metabolism (4). The inner relationship between NAFLD and thyroid hormones was then questioned and studied nowadays. Interestingly, in some clinical traials, thyroxine supplementation has been found to improve obesity and NAFLD to some extent (33, 34). High dosages of T3 were also previously used to promote weight loss in obese patients and to treat hypercholesterolemia (4). Although severe cardiac problems and loss of lean body mass prevent it from becoming a practical treatment, the finding still reinforces the validation of a potentially close relationship between thyroid hormones and metabolic disorders such as NAFLD. Several published meta-analyses (9, 10, 12) and clinical studies (15, 33, 35, 36) reached the conclusion that hypothyroidism strongly associated with NAFLD. A cross-sectional study from Korea (37), of which subjects were classified into subclinical hypothyroidism and overt hypothyroidism according to their TSH, T4, and FT4 levels, found that the spectrum of hypothyroidism correlated with NAFLD in a dose-dependent manner. This finding was consistent with a study from China (38), which suggested that even within the reference range, the gradual increase in serum FT4 level accompanied with reduced prevalence of NAFLD.

Our study also supported the notion that the risk of NAFLD increased with decreasing FT4 in a dose-dependent way. However, our analysis result does not fit perfectly with the assumption that strong association exists between hypothyroidism and NAFLD risk. Another study from Germany (39) came up with a similar result that even though serum TSH and FT3 concentrations were inconsistently associated with hepatic steatosis, low FT4 concentrations were significantly associated with hepatic steatosis. We did not find a clear dose-dependent relationship between TSH and NAFLD, and high levels of FT3 were associated with higher risk of NAFLD. It is possible that polymorphisms in the TSH receptor affect the plasma TSH to thyroid hormone ratio (40), genetic determinants, and aging can alter the set point of the TSH-FT4 feedback mechanism, resulting in a weaker TSH-FT4 association (15). The overall meta-analysis of TSH on the degree of liver fibrosis in NAFLD conducted by us found that individuals with higher levels of TSH had more significant liver fibrosis, which may confirm the relationship between TSH and FT4, and is also in line with other clinical studies. Martínez-Escudé et al. (41) found that TSH could be an independent risk factor for NAFLD and liver fibrosis in the general population. The study found the FIB-4 index of patients with subclinical hypothyroidism was higher than that of subjects with normal thyroid function (42). Punekar et al. (43) found significantly higher TSH levels in patients with cirrhosis compared to controls. Therefore, it is very likely that there is a correlation between TSH levels and the severity of liver disease. However, we unfortunately did not obtain a significant dose-response relationship between TSH and liver fibrosis after integrating the results of more large observational studies.

Studies have shown that low normal FT4 levels are significantly associated with higher insulin resistance but the relationship between FT3 and various metabolic syndrome features is unclear (40). In addition, clinical pharmacists and endocrinologists generally agree that FT3 levels within the normal range are not strongly related to thyroid function. Therefore, FT3 and FT4 may not be completely synchronized, but this does not mean that FT3 not relate to NAFLD. The investigators (19, 23) found that low FT3 levels appeared to be an independent risk factor for NAFLD and advanced liver fibrosis. Physiologically, FT3 is a biologically active form of thyroid hormone that, by binding to TRβ in hepatocytes (44), reduces intrahepatic triglyceride and cholesterol content (45), as well as inflammation and oxidative stress, and restores mitochondrial function in hepatocytes (46), to prevent the onset and progression of liver fibrosis. Correspondingly, more and more researchers (20, 47, 48) have validated the FT3/FT4 ratio as a noninvasive marker of NAFLD through clinical studies. If this hypothesis is true, the clinical diagnosis and prevention of NAFLD will be further improved.

As there is a correlation between hypothyroidism and NAFLD, it’s worth exploring if there also exists a causal relationship, which could be explained by the dose-response relationship between thyroid hormone/hypothyroidism and NAFLD. This question is also important as to whether thyroid hormone can be used clinically as a serum marker of NAFLD. A recently published mendelian randomization study (49) noted a suggestive association between hypothyroidism and NAFLD. However, after adjusting for genetically predicted BMI, the association was not significant, suggesting that this association might be influenced by BMI. The association between thyroid dysfunction and NAFLD affected by BMI, dyslipidemia and other factors was also observed in the prospective cohort study conducted by Bano et al (15). In our subgroup analysis, the association between TSH and NAFLD was also changed after adjusting for age, gender and other factors. In summary, we may hypothesize that thyroid dysfunction contributes to the development and progression of NAFLD, but it may be influenced by other factors (50), which need to be considered if thyroid hormones are used to model NAFLD non-invasive diagnostic tools. Of course, it still needs to be further investigated through analyzing longer prospective studies with larger samples.

Furthermore, this meta-analysis is the first to include high-quality (NOS>6) observational studies (case-control studies, cohort studies), and to conduct two methods (overall and dose-response meta-analysis) to explore the relationship between thyroid hormones and the risk of NAFLD and liver fibrosis. Be that as it may, this study still suffers from the following shortcomings. Firstly, although we included observational studies, the prospective cohort studies were more convincing in terms of the level of evidence for evidence-based medicine. Secondly, in the included studies the investigators focused more on TSH and FT4, but not on FT3 or other thyroid peroxidase antibody titers for which the amount of data was too small to allow adequate meta-analysis. Thirdly, the small range of thyroid hormones explored in the current article fails to observe the effect of higher levels of thyroid hormones on NAFLD, as well as possible implications for the study results. Forthly, the heterogeneity of the study results cannot be ignored. Although we explored the sources of heterogeneity through subgroup and sensitivity analyses, we were still unable to explain this phenomenon well. Due to the limited number of studies, the possibility of chance (“false positives”) for “positive” results in subgroup analyses cannot be ruled out.

## Conclusion

In conclusion, this meta-analysis suggests that high FT4, FT3 levels were associated with increased NAFLD, while high TSH levels positively correlated with liver fibrosis. However, more comprehensive prospective studies with larger samples are still needed to further clarify the relationship between thyroid-related hormones and NAFLD and to achieve more clinically meaningful evidence.

## Author contributions

LX: Writing – review & editing, Writing – original draft, Visualization, Methodology, Funding acquisition, Data curation, Conceptualization. YC: Writing – original draft, Methodology, Conceptualization. JS: Writing – review & editing, Project administration, Conceptualization. RL: Writing – original draft, Software, Investigation, Data curation. FQ: Writing – original draft, Project administration, Methodology, Data curation. YZ: Writing – original draft, Investigation, Data curation. WZ: Writing – original draft, Investigation, Data curation. lY: Writing – review & editing, Funding acquisition. XZ: Writing – review & editing, Supervision, Funding acquisition, Conceptualization.

## References

[1] Younossi ZM Koenig AB Abdelatif D Fazel Y Henry L Wymer M. Global epidemiology of nonalcoholic fatty liver disease-Meta-analytic assessment of prevalence, incidence, and outcomes. Hepatology. (2016) 64:73–84. doi: 10.1002/hep.28431 26707365

[2] Friedman SL Neuschwander-Tetri BA Rinella M Sanyal AJ. Mechanisms of NAFLD development and therapeutic strategies. Nat Med. (2018) 24:908–22. doi: 10.1038/s41591-018-0104-9 PMC6553468 29967350

[3] Lindenmeyer CC McCullough AJ. The natural history of nonalcoholic fatty liverDisease-an evolving view. Clin Liver Dis. (2018) 22:11–21. doi: 10.1016/j.cld.2017.08.003 29128051 PMC6130315

[4] Sinha RA Singh BK Yen PM. Direct effects of thyroid hormones on hepatic lipid metabolism. Nat Rev Endocrinol. (2018) 14:259–69. doi: 10.1038/nrendo.2018.10 PMC6013028 29472712

[5] Nettore IC Albano L Ungaro P Colao A Macchia PE. Sunshine vitamin and thyroid. Rev Endocr Metab Disord. (2017) 18:347–54. doi: 10.1007/s11154-017-9406-3 PMC5543192 28092021

[6] Cooper DS Biondi B. Subclinical thyroid disease. Lancet. (2012) 379:1142–54. doi: 10.1016/s0140-6736(11)60276-6 22273398

[7] Ludwig U Holzner D Denzer C Greinert A Haenle MM Oeztuerk S. Subclinical and clinical hypothyroidism and non-alcoholic fatty liver disease: a cross-sectional study of a random population sample aged 18 to 65 years. BMC Endocr Disord. (2015) 15:41. doi: 10.1186/s12902-015-0030-5 26276551 PMC4536732

[8] Ritter MJ Amano I Hollenberg AN. Thyroid hormone signaling and the liver. Hepatology. (2020) 72:742–52. doi: 10.1002/hep.31296 32343421

[9] Guo Z Li M Han B Qi X. Association of non-alcoholic fatty liver disease with thyroid function: A systematic review and meta-analysis. Dig Liver Dis. (2018) 50:1153–62. doi: 10.1016/j.dld.2018.08.012 30224316

[10] He W An X Li L Shao X Li Q Yao Q. Relationship between hypothyroidism and non-alcoholic fatty liver disease: A systematic review and meta-analysis. Front Endocrinol (Lausanne). (2017) 8:335. doi: 10.3389/fendo.2017.00335 29238323 PMC5712538

[11] Mantovani A Nascimbeni F Lonardo A Zoppini G Bonora E Mantzoros CS. Association between primary hypothyroidism and nonalcoholic fatty liver disease: A systematic review and meta-analysis. Thyroid. (2018) 28:1270–84. doi: 10.1089/thy.2018.0257 30084737

[12] Jaruvongvanich V Sanguankeo A Upala S. Nonalcoholic fatty liver disease is not associated with thyroid hormone levels and hypothyroidism: A systematic review and meta-analysis. Eur Thyroid J. (2017) 6:208–15. doi: 10.1159/000454920 PMC5567115 28868261

[13] Stroup DF Berlin JA Morton SC Olkin I Williamson GD Rennie D. Meta-analysis of observational studies in epidemiology: a proposal for reporting. Meta-analysis Of Observational Studies in Epidemiology (MOOSE) group. JAMA. (2000) 283:2008–12. doi: 10.1001/jama.283.15.2008 10789670

[14] Moher D Liberati A Tetzlaff J Altman DG. Preferred reporting items for systematic reviews and meta-analyses: the PRISMA statement. PLoS Med. (2009) 6:e1000097. doi: 10.1371/journal.pmed.1000097 19621072 PMC2707599

[15] Bano A Chaker L Plompen EP Hofman A Dehghan A Franco OH. Thyroid function and the risk of nonalcoholic fatty liver disease: the Rotterdam study. J Clin Endocrinol Metab. (2016) 101:3204–11. doi: 10.1210/jc.2016-1300 27270473

[16] Lee KW Cho YK Kim BI Lee EJ Kwon HJ. Impact of hypothyroidism on development of non alcoholic fatty liver disease; 4 year retrospective cohort study. Hepatol Int. (2015) 9:S363. doi: 10.1007/s12072-015-9609-1 PMC4712165 26770926

[17] Wang C Niu Q Lv H Li Q Ma Y Tan J. Elevated TPOAb is a strong predictor of autoimmune development in patients of type 2 diabetes mellitus and non-alcoholic fatty liver disease: A case–control study. Diabetes Metab Syndrome Obes. (2020) 13:4369–78. doi: 10.2147/DMSO.S280231 PMC7678701 33235477

[18] Gu YQ Wu XH Zhang Q Liu L Meng G Wu HM. High-Normal thyroid function predicts incident nonalcoholic fatty liver disease among middle-Aged and older euthyroid subjects. Journals Gerontology Ser a-Biological Sci Med Sci. (2022) 77:197–203. doi: 10.1093/gerona/glab037 33534875

[19] Zhang YY Li JY Liu HZ. Correlation between the thyroid hormone levels and nonalcoholic fatty liver disease in type 2 diabetic patients with normal thyroid function. BMC Endocrine Disord. (2022) 22. doi: 10.1186/s12902-022-01050-2 PMC9158236 35641932

[20] Nien HC Sheu JC Chi YC Chen CL Kao JH Yang WS. One-year weight management lowers lipopolysaccharide-binding protein and its implication in meta inflammation and liver fibrosis. PLoS One. (2018) 13. doi: 10.1371/journal.pone.0207882 PMC6245791 30458048

[21] Türker F Oral A Şahin T Türker B Koçak E Ataoğlu HE. Does the FT3-to-FT4 ratio easily predict the progression of NAFLD and NASH cirrhosis? J Int Med Res. (2021) 49:3000605211056841. doi: 10.1177/03000605211056841 34763561 PMC8593317

[22] Du J Chai SB Zhao X Sun JB Zhang XM Huo &. Association between thyroid hormone levels and advanced liver fibrosis in patients with type 2 diabetes mellitus and non-alcoholic fatty liver disease. Diabetes Metab Syndrome Obesity-Targets Ther. (2021) 14:2399–406. doi: 10.2147/dmso.S313503. Du, Chai et al. 2021. PMC8165094 34079318

[23] Naguib R Fayed A Elkemary EZ Naguib H. Evaluation of thyroid function and thyroid autoimmune disease in patients with non-alcoholic fatty liver disease. Clin Exp Hepatol. (2021) 7:422–8. doi: 10.5114/ceh.2021.111169 PMC8977877 35402722

[24] Choi S Yi DY Kim SC Kang B Lee Y Lee YM. Association between non-alcoholic fatty liver disease and subclinical hypothyroidism in pediatric patients: A retrospective multicenter study from Korea. J Pediatr Gastroenterol Nutr. (2021) 72:915. doi: 10.1097/MPG.0000000000003177 PMC8144595 34032030

[25] Liu X Guo L Xiao K Zhu W Liu M Wan R. The obesity paradox for outcomes in atrial fibrillation: evidence from an exposure-effect analysis of prospective studies. Obes Rev. (2020) 21:e12970. doi: 10.1111/obr.12970 31849187

[26] Kurex Sidik JNJ. Simple heterogeneity variance estimation for meta-analysis. Appl Stat. (2005) 54:367–84. doi: 10.1111/j.1467-9876.2005.00489.x

[27] Cai S Zhou J Pan J. Estimating the sample mean and standard deviation from order statistics and sample size in meta-analysis. Stat Methods Med Res. (2021) 30:2701–19. doi: 10.1177/09622802211047348 34668458

[28] McGrath S Zhao X Steele R Thombs BD Benedetti A. Estimating the sample mean and standard deviation from commonly reported quantiles in meta-analysis. Stat Methods Med Res. (2020) 29:2520–37. doi: 10.1177/0962280219889080 PMC7390706 32292115

[29] Xu C Doi SAR. The robust error meta-regression method for dose-response meta-analysis. Int J Evid Based Healthc. (2018) 16:138–44. doi: 10.1097/XEB.0000000000000132 29251651

[30] Durrleman S Simon R. Flexible regression models with cubic splines. Stat Med. (1989) 8:551–61. doi: 10.1002/sim.4780080504 2657958

[31] Higgins JP Thompson SG. Quantifying heterogeneity in a meta-analysis. Stat Med. (2002) 21:1539–58. doi: 10.1002/sim.1186 12111919

[32] Sedgwick P Marston L. How to read a funnel plot in a meta-analysis. Bmj. (2015) 351:h4718. doi: 10.1136/bmj.h4718 26377337

[33] Xu L Ma H Miao M Li Y. Impact of subclinical hypothyroidism on the development of non-alcoholic fatty liver disease: a prospective case-control study. J Hepatol. (2012) 57:1153–4. doi: 10.1016/j.jhep.2012.05.025 22940010

[34] Singha PS Ghosh S Ghosh D. Levothyroxine and non-alcoholic fatty liver disease: A mini review. Mini Rev Med Chem. (2024) 24:128–38. doi: 10.2174/1389557523666230314113543 36918791

[35] Pagadala MR Zein CO Dasarathy S Yerian LM Lopez R McCullough AJ. Prevalence of hypothyroidism in nonalcoholic fatty liver disease. Dig Dis Sci. (2012) 57:528–34. doi: 10.1007/s10620-011-2006-2 PMC3922233 22183820

[36] Kim D Kim W Joo SK Bae JM Kim JH Ahmed A. Subclinical hypothyroidism and low-normal thyroid function are associated with nonalcoholic steatohepatitis and fibrosis. Clin Gastroenterol Hepatol. (2018) 16:123–31.e121. doi: 10.1016/j.cgh.2017.08.014 28823829

[37] Chung GE Kim D Kim W Yim JY Park MJ Kim YJ. Non-alcoholic fatty liver disease across the spectrum of hypothyroidism. J Hepatol. (2012) 57:150–6. doi: 10.1016/j.jhep.2012.02.027 22425701

[38] Xu C Xu L Yu C Miao M Li Y. Association between thyroid function and nonalcoholic fatty liver disease in euthyroid elderly Chinese. Clin Endocrinol (Oxf). (2011) 75:240–6. doi: 10.1111/cen.2011.75.issue-2 21521285

[39] Ittermann T Haring R Wallaschofski H Baumeister SE Nauck M Dörr M. Inverse association between serum free thyroxine levels and hepatic steatosis: results from the Study of Health in Pomerania. Thyroid. (2012) 22:568–74. doi: 10.1089/thy.2011.0279 PMC3358110 22574630

[40] Roos A Bakker SJ Links TP Gans RO Wolffenbuttel BH. Thyroid function is associated with components of the metabolic syndrome in euthyroid subjects. J Clin Endocrinol Metab. (2007) 92:491–6. doi: 10.1210/jc.2006-1718 17090642

[41] Martínez-Escudé A Pera G Costa-Garrido A Rodríguez L Arteaga I Expósito-Martínez C. TSH levels as an independent risk factor for NAFLD and liver fibrosis in the general population. J Clin Med. (2021) 10. doi: 10.3390/jcm10132907 PMC8267939 34209831

[42] Tahara K Akahane T Namisaki T Moriya K Kawaratani H Kaji K. Thyroid-stimulating hormone is an independent risk factor of non-alcoholic fatty liver disease. JGH Open. (2020) 4:400–4. doi: 10.1002/jgh3.12264 PMC7273701 32514444

[43] Punekar P Sharma AK Jain A. A study of thyroid dysfunction in cirrhosis of liver and correlation with severity of liver disease. Indian J Endocrinol Metab. (2018) 22:645–50. doi: 10.4103/ijem.IJEM_25_18 PMC6166553 30294575

[44] Pascual A Aranda A. Thyroid hormone receptors, cell growth and differentiation. Biochim Biophys Acta. (2013) 1830:3908–16. doi: 10.1016/j.bbagen.2012.03.012 22484490

[45] Perra A Simbula G Simbula M Pibiri M Kowalik MA Sulas P. Thyroid hormone (T3) and TRbeta agonist GC-1 inhibit/reverse nonalcoholic fatty liver in rats. FASEB J. (2008) 22:2981–9. doi: 10.1096/fj.08-108464 18434432

[46] Sinha RA Yen PM. Thyroid hormone-mediated autophagy and mitochondrial turnover in NAFLD. Cell Biosci. (2016) 6:46. doi: 10.1186/s13578-016-0113-7 27437098 PMC4950712

[47] Gökmen FY Ahbab S Ataoğlu HE Türker B Çetin F Türker F. FT3/FT4 ratio predicts non-alcoholic fatty liver disease independent of metabolic parameters in patients with euthyroidism and hypothyroidism. Clinics (Sao Paulo). (2016) 71:221–5. doi: 10.6061/clinics/2016(04)08 PMC4825197 27166773

[48] Lai S Li J Wang Z Wang W Guan H. Sensitivity to thyroid hormone indices are closely associated with NAFLD. Front Endocrinol (Lausanne). (2021) 12:766419. doi: 10.3389/fendo.2021.766419 34803928 PMC8602917

[49] Xie J Huang H Liu Z Li Y Yu C Xu L. The associations between modifiable risk factors and nonalcoholic fatty liver disease: A comprehensive Mendelian randomization study. Hepatology. (2023) 77:949–64. doi: 10.1002/hep.32728 35971878

[50] Mishra A Panda B Ghosh D. The pathophysiology and management of NAFDL in post-menopausal women: an updated short review. OBM Geriatrics. (2023) 7:255. doi: 10.21926/obm.geriatr.2304255

