# Supplementary material for: Association between thyroid function and nonalcoholic fatty liver disease: a dose-response meta-analysis
Source: Front Endocrinol (Lausanne). 2024 Jun 25;15:1399517. doi: 10.3389/fendo.2024.1399517 (PMC11231071; doi:10.3389/fendo.2024.1399517)

***Additional file***

**Additional file1-1.** The Meta-analysis of Observational Studies in Epidemiology (MOOSE) reporting guideline

**Additional file1-2.** The Preferred Reporting Items for a Systematic Review and Meta-analysis (PRISMA) guideline

**Additional file2.** Search Strategy

**Additional file3-1.** The Newcastle Ottawa Quality Assessment Scale - Case control study

**Additional file3-2.** The Newcastle Ottawa Quality Assessment Scale - Cohort study

**Additional file4.** Calculated results of the dose-response meta-analysis (FT4 and risk of NAFLD)

**Additional file5.** The results were stable by omitting one study at once

**Additional file1-1. The Meta-analysis of Observational Studies in Epidemiology (MOOSE) reporting guideline**

| **Item No** | **Recommendation** | **Location where item is reported** |
| --- | --- | --- |
| **Reporting of background should include** | | |
| 1 | Problem definition | Introduction, Paragraph 4 |
| 2 | Hypothesis statement | Introduction, Paragraph 4 |
| 3 | Description of study outcome(s) | Introduction, Paragraph 4 |
| 4 | Type of exposure or intervention used | NA |
| 5 | Type of study designs used | Introduction, Paragraph 4 |
| 6 | Study population | Introduction, Paragraph 4 |
| **Reporting of search strategy should include** | | |
| 7 | Qualifications of searchers (eg, librarians and investigators) | Methods, Paragraph 2 |
| 8 | Search strategy, including time period included in the synthesis and keywords | Methods, Paragraph 2, Additional file2 |
| 9 | Effort to include all available studies, including contact with authors | Methods, Paragraph 5 |
| 10 | Databases and registries searched | Methods, Paragraph 1, 2 |
| 11 | Search software used, name and version, including special features used (eg, explosion) | NA |
| 12 | Use of hand searching (eg, reference lists of obtained articles) | Methods, Paragraph 2 |
| 13 | List of citations located and those excluded, including justification | Figure 1 |
| 14 | Method of addressing articles published in languages other than English | NA |
| 15 | Method of handling abstracts and unpublished studies | NA |
| 16 | Description of any contact with authors | NA |
| **Reporting of methods should include** | | |
| 17 | Description of relevance or appropriateness of studies assembled for assessing the hypothesis to be tested | NA |
| 18 | Rationale for the selection and coding of data (eg, sound clinical principles or convenience) | Methods, Paragraph 5 |
| 19 | Documentation of how data were classified and coded (eg, mutiple raters, blinding, and interrater reliability) | Methods, Paragraph 5 |
| 20 | Assessment of confounding (eg, comparability of cases and controls in studies where appropriate) | Methods, Paragraph 6 |
| 21 | Assessment of study quality, indluding blinding of quality assessors; stratifcation or regression on possible predictors of study results | Methods, Paragraph 6 |
| 22 | Assessment of heterogeneity | Methods, Paragraph 8, 10 |
| 23 | Description of statistical methods (eg, justification of whether the chosen models account for predictors of study results, dose-response models, or cumulative meta-analysis) in sufficient detail to be replicated | Methods, Paragraph 7, 8, 9, 10 |
| 24 | Provision of appropriate tables and graphics | Table 1, 2; Figure 1, 2, 3, 4, 5, 6 |
| **Reporting of results should include** | | |
| 25 | Graphic summarizing individual study estimates and overall estimate | Table 1, 2; Figure 1, 2, 3, 4, 5, 6 |
| 26 | Table giving descriptive information for each study included | Table 1, 2 |
| 27 | Results of sensitivity testing (eg, subgroup analysis) | Results, Paragraph 8, Figure 6 |
| 28 | Indication of statistical uncertainty of findings | Discussion, paragraph 5 |
| **Reporting of discussion should include** | | |
| 29 | Quantitative assessment of bias (eg, publication bias) | Results, Paragraph 2, 3, 5 |
| 30 | Justification for exclusion (eg, exclusion of non-English-language citations) | Methods Paragraph 4, Figure 1 |
| 31 | Assessment of quality of included studies | Results, Paragraph 2, 3 |
| **Reporting of conclusions should include** | | |
| 32 | Consideration of alternative explanations for observed results | Discussion, paragraph 2, 3, 4 |
| 33 | Generalization of the conclusions (ie, appropriate for the data presented and within the domain of the literature review) | Discussion, paragraph 6 |
| 34 | Guidelines for future research | Discussion, paragraph 6 |
| 35 | Disclosure of funding source | Funding |

**Additional file1-2.** The Preferred Reporting Items for a Systematic Review and Meta-analysis (PRISMA) guideline

| **Section and Topic** | **Item #** | **Checklist item** | **Location where item is reported** |
| --- | --- | --- | --- |
| **TITLE** | | |  |
| **Title** | 1 | Identify the report as a systematic review. | Title |
| **ABSTRACT** | | |  |
| **Abstract** | 2 | See the PRISMA 2020 for Abstracts checklist. | Abstract |
| **INTRODUCTION** | | |  |
| **Rationale** | 3 | Describe the rationale for the review in the context of existing knowledge. | Introduction,  Paragraph 1, 2, 3 |
| **Objectives** | 4 | Provide an explicit statement of the objective(s) or question(s) the review addresses. | Introduction,  paragraph 4 |
| **METHODS** | | |  |
| **Eligibility criteria** | 5 | Specify the inclusion and exclusion criteria for the review and how studies were grouped for the syntheses. | Methods,  paragraph 3, 4 |
| **Information sources** | 6 | Specify all databases, registers, websites, organisations, reference lists and other sources searched or consulted to identify studies. Specify the date when each source was last searched or consulted. | Methods,  paragraph 2 |
| **Search strategy** | 7 | Present the full search strategies for all databases, registers and websites, including any filters and limits used. | Figure 1 |
| **Selection process** | 8 | Specify the methods used to decide whether a study met the inclusion criteria of the review, including how many reviewers screened each record and each report retrieved, whether they worked independently, and if applicable, details of automation tools used in the process. | Methods,  paragraph 5 |
| **Data collection process** | 9 | Specify the methods used to collect data from reports, including how many reviewers collected data from each report, whether they worked independently, any processes for obtaining or confirming data from study investigators, and if applicable, details of automation tools used in the process. | Methods,  paragraph 5 |
| **Data items** | 10a | List and define all outcomes for which data were sought. Specify whether all results that were compatible with each outcome domain in each study were sought (e.g. for all measures, time points, analyses), and if not, the methods used to decide which results to collect. | Methods,  paragraph 5 |
|  | 10b | List and define all other variables for which data were sought (e.g. participant and intervention characteristics, funding sources). Describe any assumptions made about any missing or unclear information. | Methods,  paragraph 5 |
| **Study risk of bias assessment** | 11 | Specify the methods used to assess risk of bias in the included studies, including details of the tool(s) used, how many reviewers assessed each study and whether they worked independently, and if applicable, details of automation tools used in the process. | Methods,  paragraph 6 |
| **Effect measures** | 12 | Specify for each outcome the effect measure(s) (e.g. risk ratio, mean difference) used in the synthesis or presentation of results. | Methods,  paragraph 7 |
| **Synthesis methods** | 13a | Describe the processes used to decide which studies were eligible for each synthesis (e.g. tabulating the study intervention characteristics and comparing against the planned groups for each synthesis (item #5)). | Methods,  paragraph 3, 4 |
|  | 13b | Describe any methods required to prepare the data for presentation or synthesis, such as handling of missing summary statistics, or data conversions. | Methods,  paragraph 6 |
|  | 13c | Describe any methods used to tabulate or visually display results of individual studies and syntheses. | Table 1, 2 |
|  | 13d | Describe any methods used to synthesize results and provide a rationale for the choice(s). If meta-analysis was performed, describe the model(s), method(s) to identify the presence and extent of statistical heterogeneity, and software package(s) used. | Methods,  paragraph 7, 8, 9, 10, 11, 12 |
|  | 13e | Describe any methods used to explore possible causes of heterogeneity among study results (e.g. subgroup analysis). | Methods,  paragraph 8 |
|  | 13f | Describe any sensitivity analyses conducted to assess robustness of the synthesized results. | Methods,  paragraph 11 |
| **Reporting bias assessment** | 14 | Describe any methods used to assess risk of bias due to missing results in a synthesis (arising from reporting biases). | NA |
| **Certainty assessment** | 15 | Describe any methods used to assess certainty (or confidence) in the body of evidence for an outcome. | Methods,  paragraph 12 |
| **RESULTS** | | |  |
| **Study selection** | 16a | Describe the results of the search and selection process, from the number of records identified in the search to the number of studies included in the review, ideally using a flow diagram. | Results,  paragraph 1  Figure 1 |
|  | 16b | Cite studies that might appear to meet the inclusion criteria, but which were excluded, and explain why they were excluded. | Figure 1 |
| **Study characteristics** | 17 | Cite each included study and present its characteristics. | Results,  paragraph 2, 3  Table 1, 2 |
| **Risk of bias in studies** | 18 | Present assessments of risk of bias for each included study. | NA |
| **Results of individual studies** | 19 | For all outcomes, present, for each study: (a) summary statistics for each group (where appropriate) and (b) an effect estimate and its precision (e.g. confidence/credible interval), ideally using structured tables or plots. | Results,  paragraph 4, 5, 6, 7, 8  Figure 2, 3, 4, 5, 6 |
| **Results of syntheses** | 20a | For each synthesis, briefly summarise the characteristics and risk of bias among contributing studies. | Results,  paragraph 4, 5, 6, 7, 8  Figure 2, 3, 4, 5, 6 |
|  | 20b | Present results of all statistical syntheses conducted. If meta-analysis was done, present for each the summary estimate and its precision (e.g. confidence/credible interval) and measures of statistical heterogeneity. If comparing groups, describe the direction of the effect. | Results,  paragraph 4, 5, 6, 7, 8  Figure 2, 3, 4, 5, 6 |
|  | 20c | Present results of all investigations of possible causes of heterogeneity among study results. | Results,  paragraph 8 |
|  | 20d | Present results of all sensitivity analyses conducted to assess the robustness of the synthesized results. | Results,  paragraph 8 |
| **Reporting biases** | 21 | Present assessments of risk of bias due to missing results (arising from reporting biases) for each synthesis assessed. | NA |
| **Certainty of evidence** | 22 | Present assessments of certainty (or confidence) in the body of evidence for each outcome assessed. | Results,  paragraph 4, 5, 6, 7, 8  Figure 2, 3, 4, 5, 6 |
| **DISCUSSION** | | |  |
| **Discussion** | 23a | Provide a general interpretation of the results in the context of other evidence. | Discussion,  paragraph 1, 2, 3, 4 |
|  | 23b | Discuss any limitations of the evidence included in the review. | Discussion,  paragraph 5 |
|  | 23c | Discuss any limitations of the review processes used. | Discussion,  paragraph 5 |
|  | 23d | Discuss implications of the results for practice, policy, and future research. | Discussion,  paragraph 4, 6 |
| **OTHER INFORMATION** | | |  |
| **Registration and protocol** | 24a | Provide registration information for the review, including register name and registration number, or state that the review was not registered. | Methods,  paragraph 1 |
|  | 24b | Indicate where the review protocol can be accessed, or state that a protocol was not prepared. | Methods,  paragraph 1 |
|  | 24c | Describe and explain any amendments to information provided at registration or in the protocol. | NA |
| **Support** | 25 | Describe sources of financial or non-financial support for the review, and the role of the funders or sponsors in the review. | Funding |
| **Competing interests** | 26 | Declare any competing interests of review authors. | Conflict of Interest |
| **Availability of data, code and other materials** | 27 | Report which of the following are publicly available and where they can be found: template data collection forms; data extracted from included studies; data used for all analyses; analytic code; any other materials used in the review. | Data availability statement |

*Environment International* modified PRISMA report adapted from: Moher D, Liberati A, Tetzlaff J, Altman DG, The PRISMA Group (2009). Preferred Reporting Items for Systematic Reviews and Meta-Analyses: The PRISMA Statement. PLoS Med 6(7): e1000097. doi:10.1371/journal.pmed1000097. (Changes are minor, with text edits to accommodate the subject matter of the journal and formatting to fit page.)

**Additional file2.** Search Strategy

| **Database** | **Search strategy** | | **Results** |
| --- | --- | --- | --- |
| **1)PubMed**  **(To February 16, 2023)** | #1 | "Hyperthyroidism"[MeSH Terms] OR "Hypothyroidism"[MeSH Terms] OR "Thyroid Hormones"[MeSH Terms] OR "Thyrotropin"[MeSH Terms] OR"Thyroid Function Tests"[MeSH Terms] OR "subclinical hyperthyroidism"[Title/Abstract] OR "subclinical hypothyroidism"[Title/Abstract] OR "subclinical thyroid dysfunction"[Title/Abstract] | 157,129 |
|  | #2 | "Hyperthyroid*"[Title/Abstract] OR "Primary Hyperthyroidism"[Title/Abstract] OR "Hypothyroidisms"[Title/Abstract] OR "feline hyperthyroidism"[Title/Abstract] OR "hyperthyreosis"[Title/Abstract] OR "hyperthyroid function"[Title/Abstract] OR "acute hypothyroidism"[Title/Abstract] OR "thyroid deficiency"[Title/Abstract] OR "thyroid gland failure"[Title/Abstract] OR "thyroid insufficiency"[Title/Abstract] OR "actyron"[Title/Abstract] OR "Primary Hypothyroidism*"[Title/Abstract] OR "TSH Deficiency"[Title/Abstract] OR "thyroid agent"[Title/Abstract] OR "dermathycin"[Title/Abstract] OR "pretiron"[Title/Abstract] OR "Secondary Hypothyroidism*"[Title/Abstract] OR "thyropar"[Title/Abstract] OR "thyrotropar"[Title/Abstract] OR "Central Hypothyroidism*"[Title/Abstract] OR "Thyroid Hormone"[Title/Abstract] OR"TSH (Thyroid Stimulating Hormone)"[Title/Abstract] OR "Thyroid-Stimulating Hormone"[Title/Abstract] OR "Thyroid Stimulating Hormone"[Title/Abstract] OR "Thyreotropin"[Title/Abstract] OR "thyroid function analysis"[Title/Abstract] OR "Thyroid Function Test"[Title/Abstract] OR "Thyroid Gland Function Tests"[Title/Abstract] OR "Thyrotroph Thyroid Hormone Sensitivity Index"[Title/Abstract] OR "Sum Activity of Peripheral Deiodinases"[Title/Abstract] OR "SPINA-GD"[Title/Abstract] OR "Jostel's TSH Index"[Title/Abstract] OR "TSH Index"[Title/Abstract] OR "Jostel's"[Title/Abstract] OR "Jostel's Thyrotropin Index"[Title/Abstract] OR "Protein Bound Iodine Test"[Title/Abstract] OR "Secretory Capacity of the Thyroid Gland"[Title/Abstract] OR "SPINA-GT"[Title/Abstract] | 69,841 |
|  | #3 | #1 OR #2 | 174,823 |
|  | #4 | “Non-alcoholic Fatty Liver Disease” [MeSH Terms] OR “nonalcoholic steatohepatitis” [MeSH Terms] OR “nonalcoholic fatty liver disease” [Title/Abstract] OR “nonalcoholic fatty liver disease*” [Title/Abstract] OR “NAFLD” [Title/Abstract] OR “nonalcoholic steatohepatiti*” [Title/Abstract] OR “NASH” [Title/Abstract] OR"non-alcoholic fatty liver"[Title/Abstract] OR "non alcoholic fatty liver"[Title/Abstract] OR "nonalcoholic fatty liver"[Title/Abstract] OR "non-alcoholic FLD"[Title/Abstract] OR "nonalcoholic FLD"[Title/Abstract] OR "non-alcoholic steatohepatitis"[Title/Abstract] OR "non alcoholic steatohepatitis"[Title/Abstract] OR "non-alcohol steato-hepatitis"[Title/Abstract] OR "nonalcoholic fatty liver inflammation"[Title/Abstract] OR "nonalcoholic steatosis hepatitis"[Title/Abstract] OR "Nonalcoholic Fatty Liver Disease Activity Score"[Title/Abstract] | 41,370 |
|  | #5 | #3 AND #4 | 264 |
| **2) Embase**  **(To February 16, 2023)** | #1 | 'hyperthyroidism'/exp OR 'hypothyroidism'/exp OR 'thyroid hormone'/exp OR 'thyrotropin'/exp OR 'thyroid function test'/exp | 223,900 |
|  | #2 | 'subclinical hyperthyroidism':ti,ab,kw OR 'subclinical hypothyroidism':ti,ab,kw OR 'subclinical thyroid dysfunction':ti,ab,kw OR 'hyperthyroid*':ti,ab,kw OR 'primary hyperthyroidism':ti,ab,kw OR 'hypothyroidisms':ti,ab,kw OR 'primary hypothyroidism*':ti,ab,kw OR 'tsh deficiency':ti,ab,kw OR 'secondary hypothyroidism*':ti,ab,kw OR 'central hypothyroidism*':ti,ab,kw OR 'thyroid hormone':ti,ab,kw OR 'tsh:ti,ab,kw' OR 'thyroid stimulating hormone':ti,ab,kw OR 'thyroid-stimulating hormone':ti,ab,kw OR 'thyroid stimulating hormone':ti,ab,kw OR 'thyreotropin':ti,ab,kw OR 'thyroid function test':ti,ab,kw OR 'thyroid gland function tests':ti,ab,kw OR 'thyrotroph thyroid hormone sensitivity index':ti,ab,kw OR 'sum activity of peripheral deiodinases':ti,ab,kw OR 'spina gd':ti,ab,kw OR 'jostels tsh index':ti,ab,kw OR 'tsh index':ti,ab,kw OR 'jostels':ti,ab,kw OR 'jostels thyrotropin index':ti,ab,kw OR 'protein bound iodine test':ti,ab,kw OR 'secretory capacity of the thyroid gland':ti,ab,kw OR 'spina gt':ti,ab,kw | [90,579](https://www.embase.com/) |
|  | #3 | #1 OR #2 | 239,474 |
|  | #4 | 'nonalcoholic fatty liver'/exp OR 'nonalcoholic steatohepatitis'/exp | [63,557](https://www.embase.com/) |
|  | #5 | 'nonalcoholic fatty liver disease activity score':ti,ab,kw OR 'nonalcoholic fatty liver disease':ti,ab,kw OR 'nonalcoholic fatty liver disease*':ti,ab,kw OR 'nafld':ti,ab,kw OR 'nonalcoholic steatohepatiti*':ti,ab,kw OR 'nash':ti,ab,kw OR 'non-alcoholic fatty liver':ti,ab,kw OR 'non alcoholic fatty liver':ti,ab,kw OR 'nonalcoholic fatty liver':ti,ab,kw OR 'non-alcoholic steatohepatitis':ti,ab,kw OR 'non alcoholic steatohepatitis':ti,ab,kw | [66,615](https://www.embase.com/) |
|  | #6 | #4 OR #5 | [77,362](https://www.embase.com/) |
|  | #7 | #3 AND #6 | 986 |
| **3) Cochrane Library**  **(To February 16, 2023)** | #1 | MeSH descriptor: [Hyperthyroidism] explode all trees OR MeSH descriptor: [Hypothyroidism] explode all trees OR MeSH descriptor: [Thyrotropin] explode all trees OR MeSH descriptor: [Thyroid Hormones] explode all trees OR MeSH descriptor: [Thyroid Function Tests] explode all trees | 3,322 |
|  | #2 | (subclinical hyperthyroidism OR subclinical hypothyroidism OR subclinical thyroid dysfunction OR hyperthyroid OR primary hyperthyroidism OR hypothyroidisms OR primary hypothyroidism OR tsh deficiency OR secondary hypothyroidism OR central hypothyroidism OR thyroid hormone OR tsh OR'thyroid stimulating hormone OR thyroid-stimulating hormone OR thyroid stimulating hormone OR thyreotropin OR thyroid function test OR thyroid gland function tests OR thyrotroph thyroid hormone sensitivity index OR sum activity of peripheral deiodinases OR spina gd OR jostels tsh index OR tsh index OR jostels OR jostels thyrotropin index OR protein bound iodine test OR secretory capacity of the thyroid gland OR spina gt):ti,ab,kw | 6,767 |
|  | #3 | #1 or #2 | 8,116 |
|  | #4 | MeSH descriptor: [nonalcoholic fatty liver] explode all trees | 1,511 |
|  | #5 | (Non-alcoholic Fatty Liver Disease OR nonalcoholic steatohepatitis OR nonalcoholic fatty liver disease OR nonalcoholic fatty liver disease* OR NAFLD OR nonalcoholic steatohepatiti* OR NASH OR non-alcoholic fatty liver OR non alcoholic fatty liver OR nonalcoholic fatty liver OR non-alcoholic FLD OR nonalcoholic FLD OR non-alcoholic steatohepatitis OR non alcoholic steatohepatitis OR non-alcohol steato-hepatitis OR nonalcoholic fatty liver inflammation OR nonalcoholic steatosis hepatitis OR Nonalcoholic Fatty Liver Disease Activity Score):ti,ab,kw | 4,504 |
|  | #6 | #4 OR #5 | 4,504 |
|  | #7 | #3 AND #6 | 83 |
| **4) Web of science**  **(To February 16, 2023)** | #1 | TS= (Hyperthyroidism OR Hypothyroidism OR Thyroid Hormones OR Thyrotropin OR Thyroid Function Tests OR subclinical hypothyroidism OR subclinical hyperthyroidism OR subclinical thyroid dysfunction) | 66,198 |
|  | #2 | TS= (Non-alcoholic Fatty Liver Disease OR nonalcoholic fatty liver disease OR fatty liver OR nonalcoholic steatohepatiti* OR NASH OR steatohepatiti* OR nonalcoholic fatty liver disease* OR fatty liver* OR NAFLD OR liversteatos*) | 114,962 |
|  | #3 | #1 AND #2 | 851 |

**Additional file3-1.** The Newcastle Ottawa Quality Assessment Scale - Case control study

Note: A study can be awarded a maximum of one star for each numbered item within the Selection and Exposure categories. A maximum of two stars can be given for Comparability.

Selection

1) Is the case definition adequate?

a) yes, with independent validation ☆ 

b) yes, eg record linkage or based on self reports

c) no description

2) Representativeness of the cases

a) consecutive or obviously representative series of cases ☆

b) potential for selection biases or not stated

3) Selection of Controls

a) community controls ☆ 

b) hospital controls

c) no description

4) Definition of Controls

a) no history of disease (endpoint) ☆ 

b) no description of source

Comparability

1) Comparability of cases and controls on the basis of the design or analysis

a) study controls for _______________ (Select the most important factor.) ☆ 

b) study controls for any additional factor (This criteria could be modified to indicate specific control for a second important factor.) ☆

Exposure

1) Ascertainment of exposure

a) secure record (eg surgical records) ☆ 

b) structured interview where blind to case/control status ☆ 

c) interview not blinded to case/control status

d) written self report or medical record only

e) no description

2) Same method of ascertainment for cases and controls

a) yes ☆ 

b) no

3) Non-Response rate

a) same rate for both groups ☆ 

b) non respondents described

c) rate different and no designation

**Additional file3-2.** The Newcastle Ottawa Quality Assessment Scale - Cohort study

Note: A study can be awarded a maximum of one star for each numbered item within the Selection and Outcome categories. A maximum of two stars can be given for Comparability

Selection

1) Representativeness of the exposed cohort

a) truly representative of the average _______________ (describe) in the community ☆ 

b) somewhat representative of the average ______________ in the community ☆ 

c) selected group of users eg nurses, volunteers

d) no description of the derivation of the cohort

2) Selection of the non-exposed cohort

a) drawn from the same community as the exposed cohort ☆ 

b) drawn from a different source

c) no description of the derivation of the non-exposed cohort

3) Ascertainment of exposure

a) secure record (eg surgical records) ☆ 

b) structured interview ☆ 

c) written self-report

d) no description

4) Demonstration that outcome of interest was not present at start of study

a) yes ☆ 

b) no

Comparability

1) Comparability of cohorts on the basis of the design or analysis

a) study controls for _____________ (select the most important factor) ☆ 

b) study controls for any additional factor (This criteria could be modified to indicate specific control for a second important factor.) ☆

Outcome

1) Assessment of outcome

a) independent blind assessment ☆ 

b) record linkage ☆ 

c) self-report

d) no description

2) Was follow-up long enough for outcomes to occur

a) yes (select an adequate follow up period for outcome of interest) ☆ 

b) no

3) Adequacy of follow up of cohorts

a) complete follow up - all subjects accounted for ☆ 

b) subjects lost to follow up unlikely to introduce bias - small number lost - > ____ % (select an adequate %) follow up, or description provided of those lost) ☆ 

c) follow up rate < ____% (select an adequate %) and no description of those lost

d) no statement

**Additional file4.** Calculated results of the dose-response meta-analysis

The nonlinear dose-response analysis of FT4 and risk of NAFLD.

| Model | Non-linear | | | | | |
| --- | --- | --- | --- | --- | --- | --- |
| Num knots | 3 | | | | | |
| Knot values | 1, 1, 1 | | | | | |
| Num obs | 13 | | | | | |
| Num studies | 6 | | | | | |
| R-squared | 0.141 | | | | | |
| Root MSE | 0.125 | | | | | |
|  | Coef. | Robust Std. Err. | t | P>|t| | [95% Conf. Interval] | |
| _doses1 | -0.3110335 | 0.0574744 | -5.41 | 0.003 | -0.458776 | -0.1632909 |
| _doses2 | 0.6151078 | 0.1712772 | 3.59 | 0.016 | 0.1748257 | 1.05539 |
| _cons | 0.3217093 | 0.0586366 | 5.49 | 0.003 | 0.1709792 | 0.4724394 |

We took the doses of 1.019 to 1.54 ng/dL at the first spline which corresponds to 0.3237 to 1.324073 at the second spline to estimate an overall linear trend per 1 ng/dL of FT4 concentration increase as follows:

**Additional file5.** The results were stable by omitting one study at once

1. TSH-Risk of NAFLD


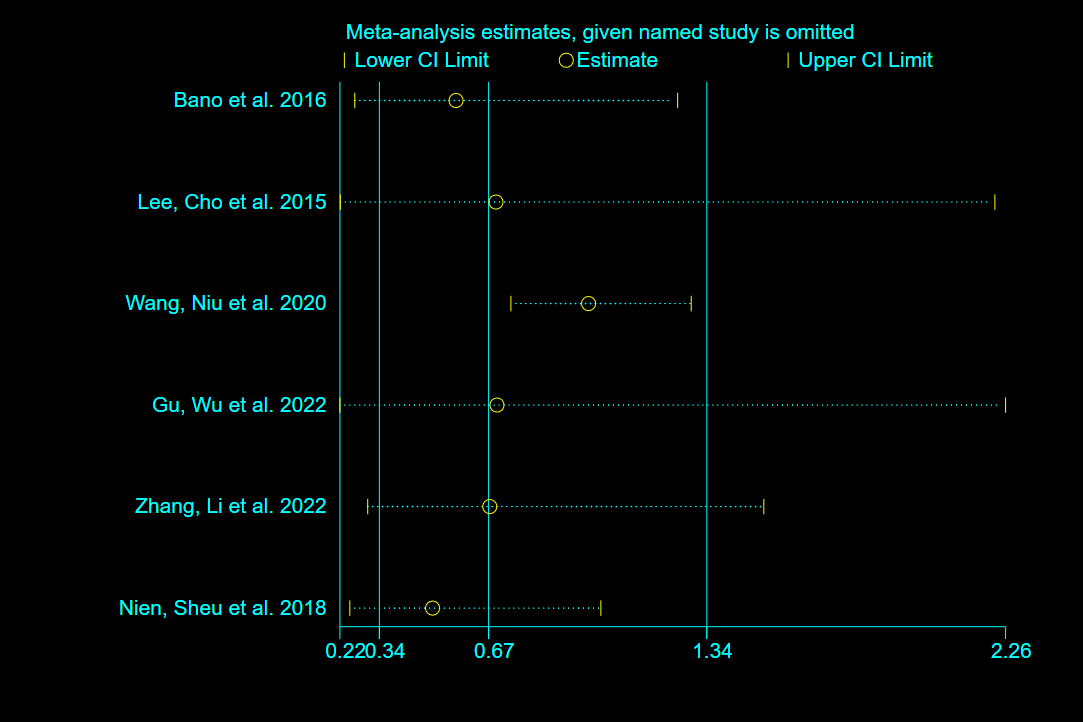


1. FT4-Risk of NAFLD


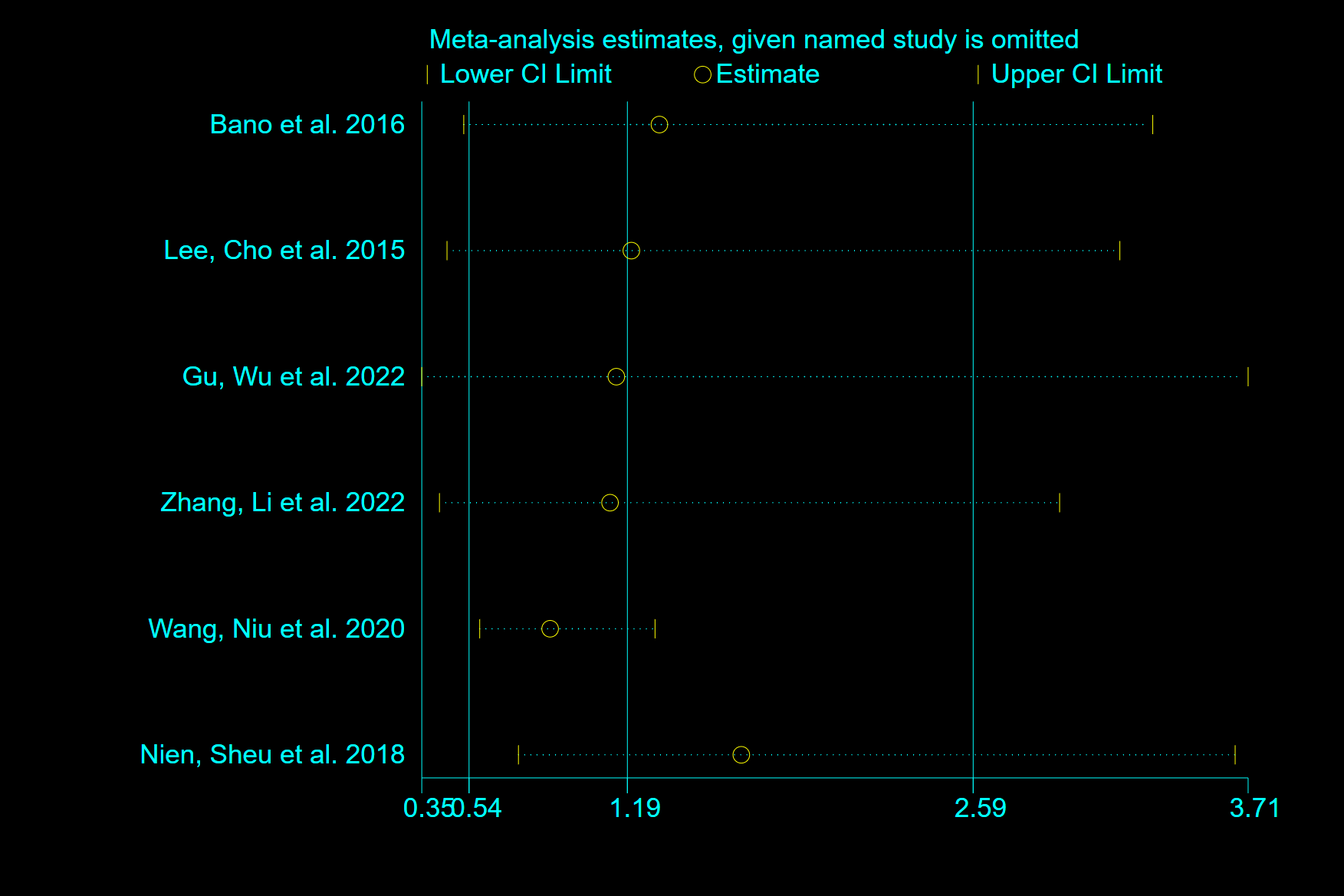


1. FT3-Risk of NAFLD


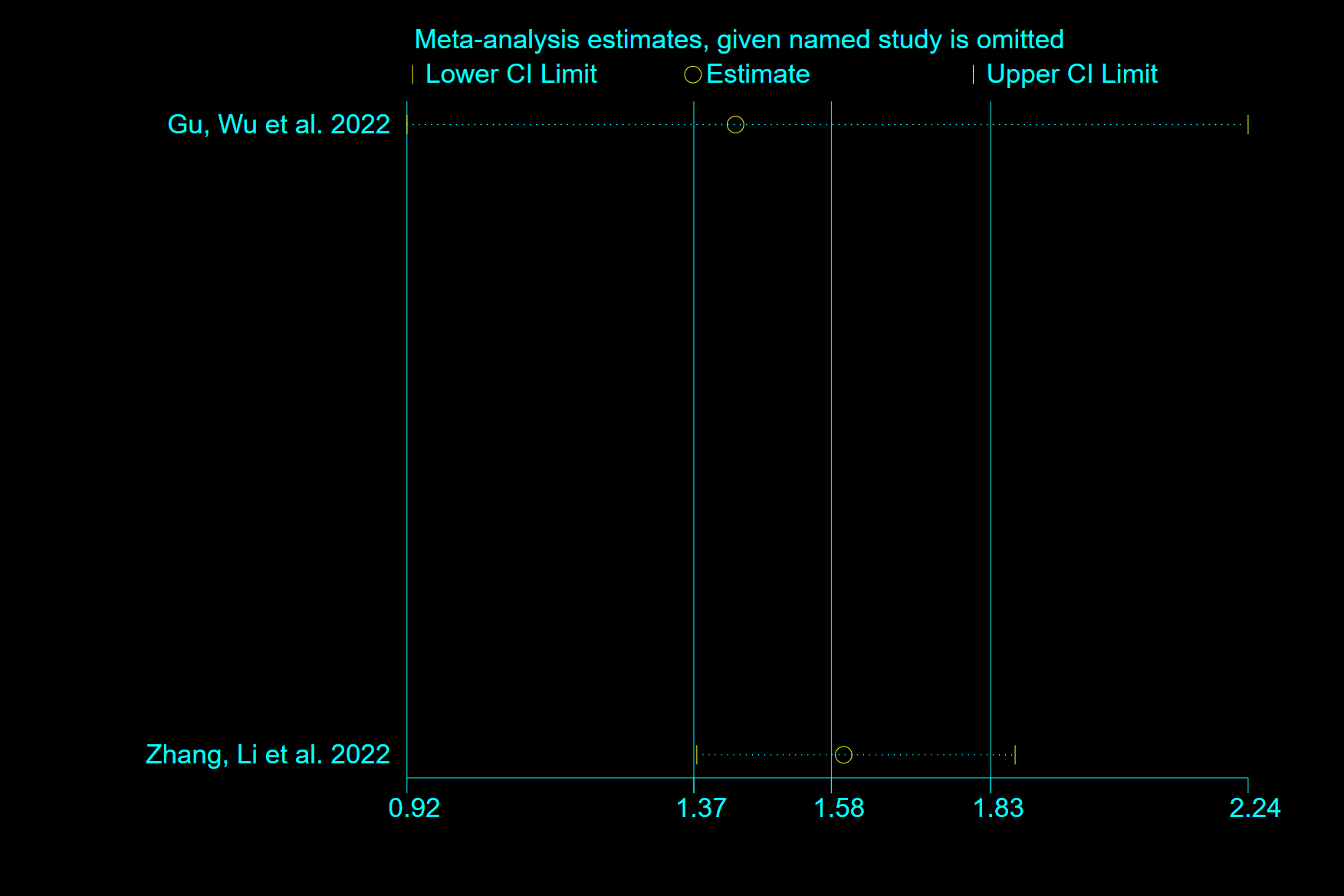


1. TSH- Liver fibrosis index of NAFLD


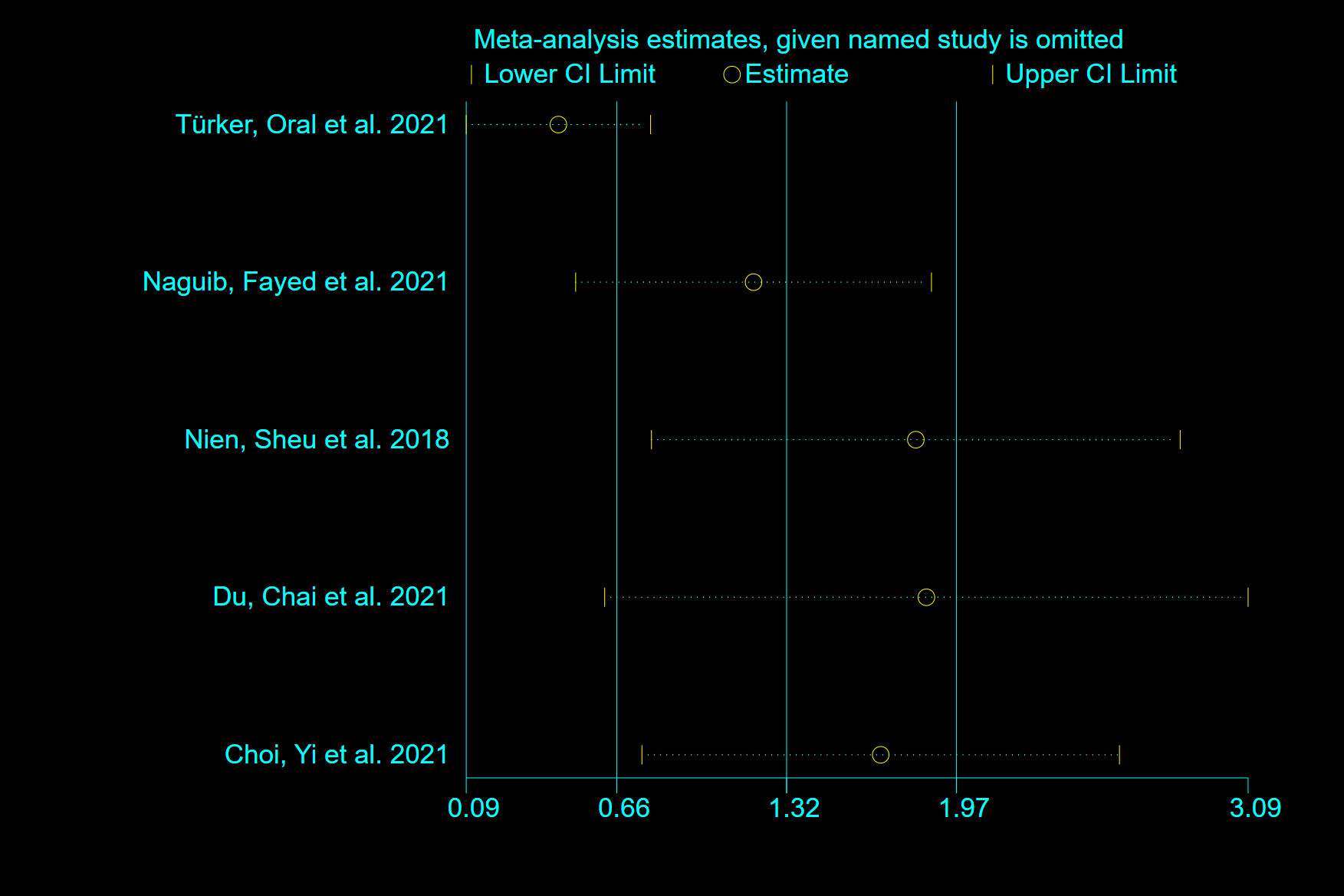


1. FT4- Liver fibrosis index of NAFLD


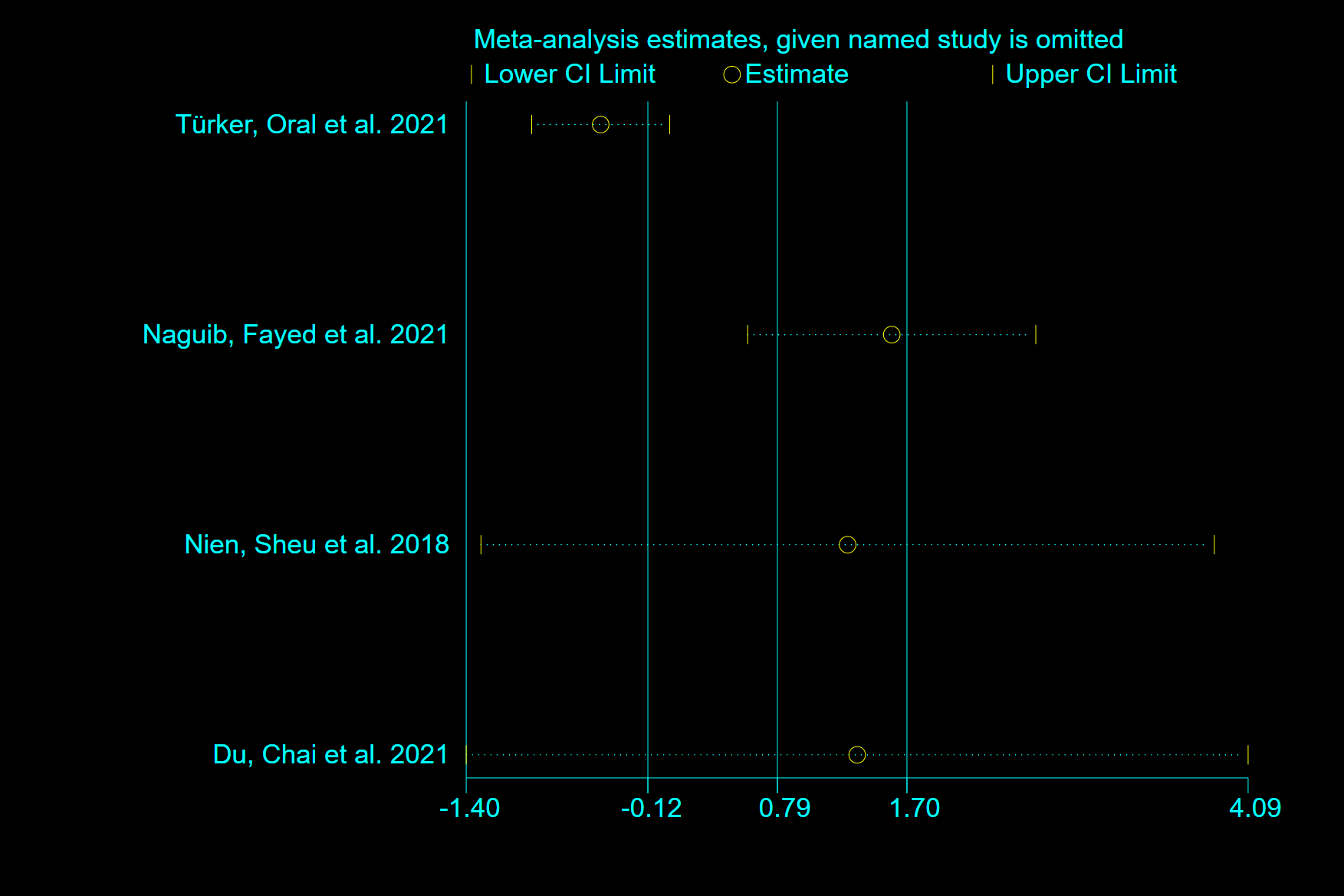


1. FT3- Liver fibrosis index of NAFLD


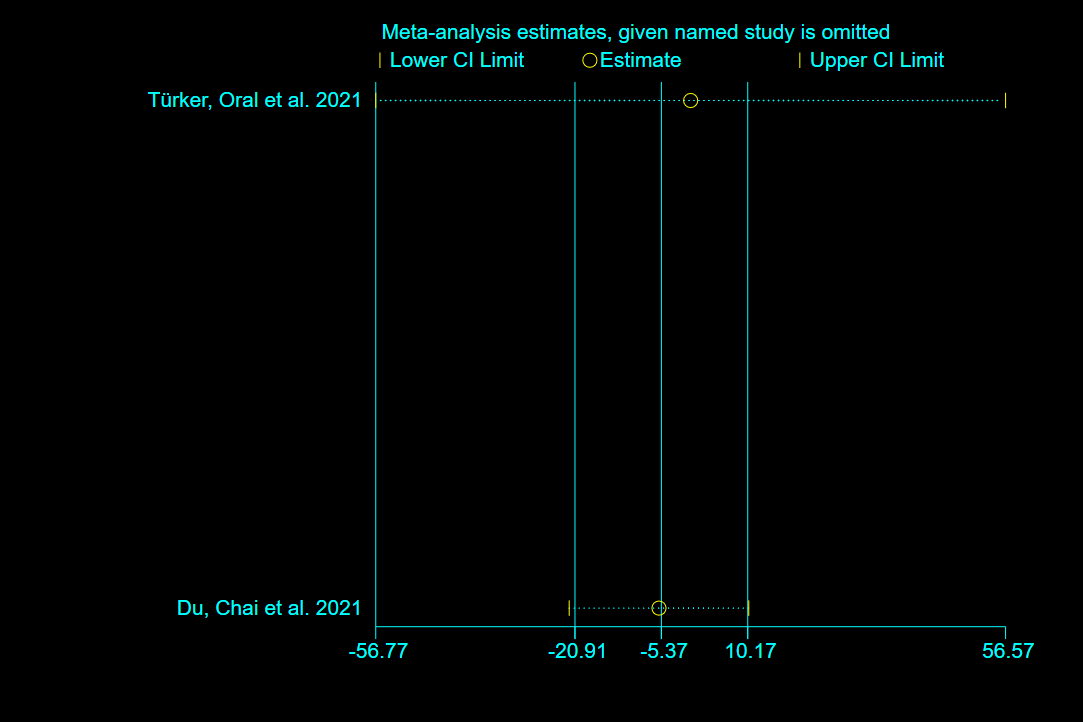

Supplement: Supplementary file 1 [file DataSheet_1.doc]
